# Assessing Pulmonary Embolisms on Unenhanced CT Images Using Electron Density Images Derived from Dual-Layer Spectral Detector CT: A Single-centre Prospective Study Conducted at the Emergency Department

**DOI:** 10.2174/0115734056316803241021102932

**Published:** 2025-01-17

**Authors:** Huayang Du, Xin Sui, Ruijie Zhao, Jiaru Wang, Ying Ming, Sirong Piao, Jinhua Wang, Xiaomei Lu, Lan Song, Wei Song

**Affiliations:** 1 Department of Radiology, Peking Union Medical College Hospital (PUMCH), Chinese Academy of Medical Sciences & Peking Union Medical College (CAMS & PUMC), Beijing, China; 2 CT Clinical Science, Philips Healthcare, Beijing 100600, China

**Keywords:** Dual-energy computed tomography, Pulmonary embolism, Electron density, Overlay electron density, Computed tomography, Pulmonary angiography

## Abstract

**Background:**

Multiple spectral images can be extrapolated from Spectral Detector CT (SDCT), ED, and OED images. ED and OED images are highly sensitive to moisture-rich tissues. Moreover, they have the potential to detect pulmonary artery thrombi in non-enhanced chest CT images.

**Objective:**

The objective of this study was to assess the sensitivity, specificity, and accuracy of ED and OED images obtained using SDCT for the detection of pulmonary embolism on non-enhanced images.

**Aims:**

This study aimed to evaluate the utility of unenhanced spectral imaging, Electron Density (ED), and Overlay Electron Density (OED) images for assessing pulmonary embolisms in patients with suspected or confirmed Acute Pulmonary Embolism (APE).

**Methods:**

Seventy-nine patients who underwent unenhanced and Computed Tomography Pulmonary Angiography (CTPA) using dual-layer spectral detector CT to evaluate APE between November, 2021 and April, 2022 were enrolled in this retrospective study. Based on unenhanced spectral and CTPA images, two radiologists identified areas of high density in the main, lobar, and segmental pulmonary arteries on ED and OED images and detected Pulmonary Embolism (PE) on enhanced images using a consultative approach. CTPA results were considered the gold standard. The diagnostic performance of ED and OED in detecting PE was analyzed.

**Results:**

PE was detected in 40 patients (40/79), and 17, 69, and 20 PEs were detected in the main, lobar, and segmental arteries, respectively. The PE detection sensitivity on ED images was 69.7–94.7%, and the specificity was 58.5–98.2% for the individual, main, lobe, and segmental pulmonary arteries. The sensitivity and specificity for OED images were 94.1–95.2% and 80.0–98.1%, respectively. The positive predictive value (PPV) and negative predictive value (NPV) were 53.6–87.7% and 69.7–95.9% for ED images and 48.5–88.9% and 94.1–98.9% for OED images, respectively. The accuracy was 76.0–98.9% and 87.3–96.2% when using ED and OED images, respectively. The research identified that whether it was main, lobar, or segmental pulmonary arteries with blood clots, EDW values ranged from 108.1–108.8%EDW, which were 3.9–4.2%EDW higher than those of arteries without emboli. Pulmonary arteries with emboli standardised ED values were 103.6-104.3%EDW.

**Conclusion:**

ED and OED images using spectral CT without contrast media demonstrated high diagnostic performance and could improve the visualization of PE.

## 1 INTRODUCTION

The incidence of pulmonary embolism accounts for 39–115 per 100,000 persons every year [1], leads to over 300,000 deaths annually in the United States, and ranks as the third most prevalent cause of cardiovascular mortality, following stroke and myocardial infarction [1, 2]. Acute Pulmonary Embolism (APE) is life-threatening when not recognized and treated in time [3, 4]. Computed Tomography Pulmonary Angiography (CTPA) is the first-line diagnostic method and the gold standard for identifying and assessing severity in patients with suspected APE in the emergency department [5]. Due to the variable and nonspecific clinical presentation of pulmonary embolisms, diagnosis can be challenging. To minimize associated morbidity and mortality, patients with suspected PE should be evaluated promptly so that appropriate therapy can be initiated as soon as possible. However, the overuse of CTPA examinations for PE remains an important medical problem, particularly in university hospitals [6, 7]. Due to the hazards of radiation exposure and contrast agents, patients should not undergo CTPA unnecessarily. About 9.4% of CTPA could potentially be avoided [8]. Chest CT scans without intravenous contrast are conducted regularly for multiple clinical applications, such as trauma, pneumonia evaluation, and nonspecific chest pain.

The hyperdense lumen observed in non-contrast CT images obtained from multidetector CT scanners may suggest the existence of blood clots in central PE [9]; however, conventional CT images can only provide differences between tissue densities. Dual-energy CT measures tissue attenuation at different energies and offers additional information in clinical practice, such as Virtual Monochromatic (VM) imaging and Iodine Concentration (IC) mapping. Spectral Detector CT (SDCT) is a unique dual-energy CT technology with a dual-layer spectral detector. Spectral Base Images (SBI) can be generated from SDCT, such as Virtual Monoenergetic Images (VMI) that assess the attenuation that would result from a monochromatic X-ray source, iodine maps, effective atomic number images, Electron Density (ED), and Overlay Electron Density (OED) images [10, 11]. In short, compared to conventional CT scanners, SDCT technology offers significant advantages for clinical diagnosis [12-14]. ED and OED images improve tissue visualization [12, 15, 16]. Previous studies have indicated the diagnostic capacity of ED images in suspected or confirmed coronavirus disease (COVID-19) [15] and the improvement in the diagnostic accuracy of post-traumatic prevertebral haematoma of the cervical spine [16]. There was only one case report on the use of unenhanced ED images in PE detection [17]. It is hypothesized that ED/OED images obtained by unenhanced CT could signal the presence of PE.

This study aimed to assess the possibilities of unenhanced chest CT spectral imaging of ED/OED images in detecting acute pulmonary embolism. Evaluating potential changes in ED/OED images caused by APE has potential benefits. The sensitivity, specificity, Negative Predictive Value (NPV), and Positive Predictive Value (PPV) of hyperdensity from OE/OED images were assessed in unenhanced CT to detect acute PE using concomitant CTPA as the gold standard.

## 2 MATERIALS AND METHODS

### 2.1 Permission

The ethics committee of our hospital reviewed this prospective study. All patients or patient responsible persons were provided comprehensive information and signed standardized informed consent forms.

### 2.2 Sample Size

Sample size calculations were performed using the PASS software (version 21.0.3) with the following settings: Alternative Hypothesis, Two-Sided (H1:ō≠0), Population Size: Infinite, Power = 0.9, Alpha = 0.0561, mean of paired differences = 4.13, and standard deviation of paired differences = 3.7. The required sample size was calculated to be 11 individuals. According to the previous studies [16], the sample was determined to be over 60 patients.

### 2.3 Patient Selection

This study recruited consecutive patients with suspected PE from the emergency department between November, 2021 and April, 2022. All patients agreed to undergo the CT examination. Patients with large pleural effusions, previous pulmonary surgery, significant breathing movement artifacts, or other conditions that may negatively impact the imaging quality were excluded. Those with severe pneumonia were also excluded, as the literature demonstrates that pneumonia obscures the pulmonary arteries and that the high density of the ED due to pneumonia can affect the accuracy of PE diagnosis [15].

### 2.4 Imaging Technique

CT examinations were conducted on a dual-layer detector spectral CT (IQon Spectral CT, Philips Healthcare, the Netherlands). The following CT imaging settings were used: a tube voltage of 120 kVp, a tube current adjusted automatically based on dose modulation (DoseRight, Philips Healthcare), and a DoseRight index of 15. Other imaging parameters were 0.27 s gantry rotation time, 0.703 pitch, 64×0.625 collimation, 1.0 mm reconstruction thickness and slice thickness, 35 cm Field of View (FOV), and 512 *×* 512 matrix. CT images were acquired during a deep inspiratory breath hold and head-to-feet orientation, and the patients' hands were lifted over their heads.

The CT scanning protocol was to perform an unenhanced chest CT first, followed by pulmonary artery enhancement, which was the routine CTPA protocol on any scanner in our hospital. Nonionic contrast media (Iopamiro, 370 mg/mL; Bracco Sine Pharma) was administered through a 20-G trocar using a dual-syringe power injector. A total of 45 ml of contrast agent was infused at a rate of 3–5 ml/s, with an additional 30 ml of saline infused simultaneously at an equivalent rate. A bolus was administered to track the Region of Interest (ROI) in the inferior vena cava, and the scan was initiated once the bolus reached the 150 Hounsfield Units (HU) threshold. Unenhanced and enhanced images were reconstructed using iDose^4^ (level 3) and spectral level 3, respectively. All images were transferred to GEPACES, and Spectral Base Image (SBI) data were transferred to a Philips workstation (IntelliSpace Portal, Version 10.1) for subjective assessment and quantitative measurement.

### 2.5 Imaging Analysis

#### 2.5.1 Subjective Assessment

The images were assessed subjectively by two radiologists with over 5 years of experience in lung investigations using dual-source imaging using a consultative method on the GEPACS system. The pulmonary artery was assessed on the enhanced images for the presence of thrombi, window width, and window level (W350, L50). Thrombi were defined as areas of reduced density within a blood vessel, either partially or completely obstructing the lumen, and were surrounded by a dense ring of enhanced blood that was seen on two or more consecutive images [18]. Radiologists were allowed to adjust the appropriate W/L as in a real clinical scenario during the assessment. Thirty arteries were evaluated in each patient's CTPA images. The detailed pulmonary arteries included the following pulmonary artery trunks: the main pulmonary artery, right and left pulmonary arteries, and pulmonary lobe arteries (right upper lobe artery, right middle lobe artery, right lower lobe artery, right middle lobe artery, left upper lobe artery, left lingula lobe artery, and left lower lobe artery). The pulmonary segmental arteries included the apical segmental artery (RA1), posterior segmental artery (RA2), anterior segmental artery (RA3), lateral segment artery (RA4), medial segment artery (RA5), superior segmental artery (RA6), medial basal segmental artery (RA7), anterior basal segmental artery (RA8), lateral basal segmental artery (RA9), posterior basal segmental artery (RA10), apical segmental pulmonary artery (LA1), posterior segmental artery (LA2), anterior segmental artery (LA3), superior lingula artery (LA4), inferior lingula artery (LA5), superior segmental artery (LA6), medial basal segmental artery (LA7), anterior basal segmental artery (LA8), lateral basal segmental artery (LA9), and posterior basal segmental artery (LA10).

After a 2-week washout period for diagnosing PE on CTPA, two trained radiologists evaluated non-enhanced ED and OED images on a Philips workstation using a consultative method. High-density pulmonary arteries on two or more consecutive ED images and pulmonary arteries with color markings on two or more consecutive OED images were recorded, representing pulmonary arteries that may contain thrombi. The anatomical location of the arteries assessed on the ED/OED images was consistent with the enhanced images.

#### 2.5.2 Quantitative Image Quality Evaluation

The mean ED values (percentage relative to the Electron Density of Water, %EDW) of the main pulmonary artery were measured as a baseline, avoiding regions of visible high density and areas of thrombus. A CTPA diagnosis was utilized as the gold standard for comparison, and the ROIs were placed in PE regions in the right and left main pulmonary arteries, as well as in each lobar and segmental artery on unenhanced ED or OED images. The ROI was set as large as possible to contain the maximum high-density area of EDW or overlay EDW. The ROIs were delineated by a qualified radiologist possessing seven years of experience. Each ROI was measured twice, and the mean value was computed to address the potential bias of quantitative evaluation.

In this study, relative Electron Density difference (∆ED) and Normalized Electron Density (NED) were introduced to account for differences in absolute values of ED between individuals based on iodine difference and Normalized Iodine Content (NIC) in the literature [19]. ∆ED and NED were calculated using the following formulas (Eqs **1** and **2**):

**Table:** 

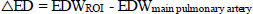	(1)

**Table:** 

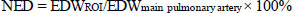	(2)

### 2.6 Statistical Analysis

The data presented in this study were presented as the mean ± standard deviation (SD) if they conformed to the normal distribution after the Kolmogorov-Smimov test or as the median with a range if they did not conform to the normal distribution. Both parametric and nonparametric tests were performed to compare values across different groups, including t-tests and χ2 tests. Additionally, the sensitivity, specificity, and positive and negative predictive values were assessed in contingency tables. A statistical significance level of *P* < 0.05 was set for all tests. Data analyses were conducted using the IBM-SPSS version 22.0 software package (IBM, USA).

## 3 RESULTS

Eighty five patients were included in the study: 1 patient underwent lung resection, 1 had a large pleural effusion, 1 had a large fluid pneumothorax, 2 patients had significant motion artefacts, and 1 patient refused to be included in the study. Excluding 6 patients, a total of 79 patients were enrolled in the study; 36 (45.57%) were men, 43 (54.43%) were women, and the mean age (52.3 ± 17.9) years) ranged from 14 to 85 years.

Enhanced results from the computed tomography pulmonary angiography showed that pulmonary embolisms were detected in 40 out of 79 patients. In total, 17 embolisms were detected in the main pulmonary arteries, 69 embolisms in the lobar arteries, and 20 in the segmental arteries. The detailed locations of the embolisms are mentioned in Table **1**.

Both ED and OED images demonstrated a high sensitivity and specificity for predicting PE in the main, lobar, and segmental levels of pulmonary arteries. The sensitivity of ED images ranged from 57.7% to 95.6%, while the specificity ranged from 89.9% to 98.8%. For OED images, the sensitivity ranged from 69.2% to 99.7%, and the specificity ranged from 87.7% to 98.8%. The PPV for detecting PE in the pulmonary artery trunks was 53.6% in ED images and 48.5% in OED images. The PPV for the detection of PE in the lobular artery and pulmonary segment artery was 67.9% and 87.7% in ED images, respectively; in OED images, it was 68.1% and 88.9% (Tables **2** and **3**).

A total of 2 patients had false-negative results on ED/OED images. Specifically, 3 PEs were missed on ED images and 1 on OED images in the main pulmonary arteries, 7 PEs on ED and 5 on OED images in the lobar arteries, and 57 PEs on ED and 35 on OED images in the segmental arteries (Table **2**). In the main pulmonary arteries, the missed PEs were charac-terized by small emboli with a maximum diameter of less than 4 mm on enhanced axial images. These emboli were located near the margins of the pulmonary arteries, where there were multiple calcifications in the adjacent arteries. In the lobar and segmental arteries, the missed PEs were also small, with a maximum diameter of less than 3 mm on enhanced CT axial images. Additionally, some of these arteries exhibited respira-tory motion artifacts.

**Fig. (1):**
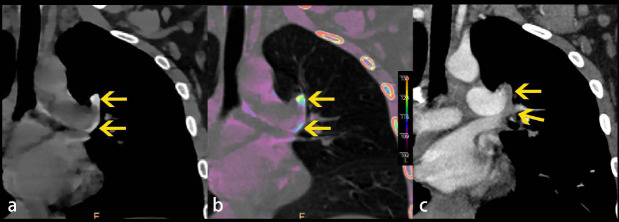
A 14-year-old pulmonary hypertension female with sudden severe chest pain. **A**, Electron density spectral CT coronal image shows high-density lesions (yellow arrow) in the margin of the left main pulmonary artery and left upper lobe artery. **B**, Overlay electron density spectral CT image shows high-density lesions with added color (yellow arrow) in the margin of the left main pulmonary artery and left upper lobe artery. **C**, Follow-up enchanted chest CT image obtained immediately after images in A and B confirming the presence of PE (yellow arrow).

**Fig. (2):**
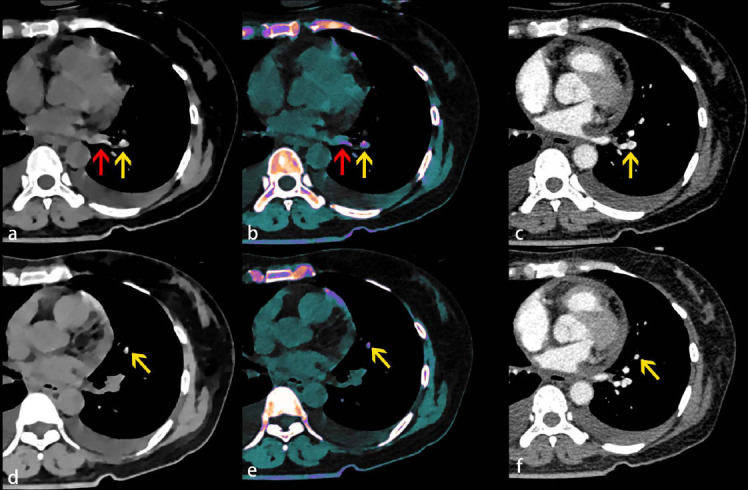
A 49-year-old female presented with abnormal coagulation and intermittent chest pain. Serum biochemistry test was as follows: prothrombin time (PT)-17.3 s(↑), PT%-49.6%(↓), international normalized ratio (INR)-1.51(↑), fibrinogen (Fib)-0.61 g/L(↓), activated partial thromboplastin time (APTT)-44.7 s (↑), activated partial thromboplastin time ratio (APTT-R)-1.65 (↑), thrombin time (TT)-28.8 s (↑), D-dimer-49.49 (↑). **A**, **D** Unenhanced electron density spectral CT axial images show high-density lesions (yellow arrow) in the left lower lobe basal segmental artery (A) and left lingula lobe artery (D). The pulmonary vein area in the lower lobe of the left lung also shows high density (A and D, red arrow). **B**, **E** Overlay electron density spectral CT axial images show high-density lesions with added color (yellow arrow) in the left lower lobe basal segmental artery (A) and left lingula lobe artery (D). **C**, **F** Enhanced chest CT images confirm the presence of clots in the area of suspected segmental artery embolisms in images A-B and D-E (yellow arrows). No blood clot in the area of the pulmonary vein was seen.

The EDW value of the main pulmonary arteries in the absence of pulmonary embolism was 104.1 ± 0.7%. The EDW of pulmonary artery trunks, lobular, and pulmonary segment pulmonary arteries with embolisms was in the range of 108.1–108.8%, which exceeded that of main pulmonary arteries without embolisms by 3.9–4.2%EDW, and the differences were all statistically significant (all *P* < 0.05). Furthermore, the normalized electron density in the main pulmonary, lobar, and segmental arteries was approximately 104.0% (ranging from 103.6 to 104.3%). NED values of segmental arteries were lower than those of lobar arteries, and the difference was statistically significant (*P* < 0.05). At the same time, there was no statistically significant difference between the remaining two groups of main pulmonary arteries, lobar arteries, and main pulmonary and segmental arteries (Table **4**, Figs. **1** and **2**).

## 4 DISCUSSION

This study investigated the capacity of unenhanced CT SBI images to detect PE of the main pulmonary arteries, lobar arteries, and segmental arteries. This study demonstrated that electron density and overlay ED images were beneficial in detecting thrombosis in the pulmonary artery in unenhanced CT images. Both ED and OED images had high negative and positive predictive values when assessing the main pulmonary arteries, lobar arteries, and segmental arteries, with a high degree of accuracy.

In conventional CT images, the CT value describes how the X-ray beam is attenuated within each voxel of body tissue and is related to the number of atoms in the tissue [19, 20]. In some typical central PE cases, conventional unenhanced CT images may reveal minor increases in attenuation compared with normal pulmonary arteries [21]. The CT value differences in blood contents and thrombus on CT images without contrast are not sufficient to indicate the presence of embolisms, and a slight increase in CT attenuation is often overlooked by diagnostic observers. As a result, unenhanced images have very low specificity and sensitivity for detecting central PE [22]. Although the negative predictive value was over 90% in central PE exclusion [22], the low sensitivity and specificity indicated that imaging without contrast agent injection was insufficient for diagnosing PE. Furthermore, even though central PE diagnosis was possible in some studies, the low sensitivity of peripheral PE detection (24–28%) [23] severely limits its application. Enhanced CT with contrast often becomes essential to confirm embolisms in patients.

CTPA is the gold standard tool for identifying and evaluating suspected or confirmed PE patients with chest pain. However, other than PE, chest pain has many other causes, such as pneumonia, pneumothorax, rib fractures, coronary stenosis, and myocardial infarction. As CT examinations increase, the question arises as to whether unenhanced images provide enough information to clinicians. The spectral CT used in this study could provide as much information as possible compared to conventional CT scanners. SDCT has one X-ray tube and a dual-layer detector [24]; the upper layer of the detector absorbs low-energy photons, and the lower layer absorbs high-energy photons [25]. The SDCT scanner acquires energy spectrum images based on a unique detector, which does not require specific acquisition protocol changes in advance and allows SBI data to be reconstructed retrospectively for each scan [26].

The ED or OED value derived from SBI data is expressed in each voxel as a percentage relative to the ED of water (expressed as %EDW) [27]. Any pathologies contributing to a slight increase in tissue water can be visualized on the ED images. ED images improve the visualization of ground glass opacities in the lungs caused by coronavirus disease (COVID-19) compared to conventional CT and can predict inflammatory changes in the lungs at an early stage [15]. SDCT with ED images makes spine haematomas easier to detect than conventional images [16, 28]. The principle of thrombosis is similar to that of haematoma, with red blood cells and fibre protein aggregation. For APE cases, ED images clearly show water rich blood clots present at a higher blood density. In this study, all blood clots had higher EDs than blood, with relative electron density value differences over 4% EDW (3.9–4.2% EDW). In OED images, different colors were added to ED greyscale images based on the density of the tissue, making the tissue more visible. Additionally, the density of pulmonary arterial blood clots was slightly higher than that of the pulmonary arterial blood, which showed a color difference on the OED images. By adjusting the OED images through the windowing technique, the color difference in the blood clots can make the plaque easier to note, observe, and identify.

For all cases with or without PE, the sensitivity for OED and ED were 94.1–95.4% and 75.7–94.7%, respectively. The segmental arteries in the ED images had a relatively low sensitivity of 75.7%, which was still higher than the sensitivity for central PE diagnosed on conventional unenhanced chest CT (66.7%) [23]. Meanwhile, PEs in all other ED image locations and all precise OED locations had a very high sensitivity. The lack of OED image color suggestive of a PE caused some cases of PE to be overlooked, which could contribute to the decreased sensitivity of pulmonary segmental arteries on ED images. The PPV of both ED and OED images was relatively low, ranging from 53.6% to 67.9% and 48.5% to 68.1%, respectively, in the central type of pulmonary artery. This observation could partially be due to the high sensitivity and haemodynamic characteristics of the pulmonary arteries. Notably, the pulmonary arterial trunk, particularly the left side, is susceptible to disruptions in blood flow, resulting in the emergence of high-density artifacts in ED images.

In this study, only two patients had false-negative results, and these cases were characterized by the following factors: the thrombi were too small to generate sufficient density on ED/OED images; they were influenced by adjacent calcified foci or calcified lymph nodes, leading to the thrombus being mistaken for a calcified high-density artifact; and the poorly defined borders of the high-density areas on ED/OED images, caused by respiratory motion, were often misinterpreted as inhomogeneous blood within the pulmonary arteries. While the total number of missed PEs seemed high, most of these missed PEs were small and located in the pulmonary segmental arteries, where they were unlikely to cause significant changes in the patient's hemodynamics or affect the choice of treatment regimen.

Non-enhanced ED/OED images have an essential role in indicating the presence of embolisms in the pulmonary arteries, with excellent performance in all aspects of sensitivity, specificity, PPV, NPV (ED > 69%, OED > 94%), and accuracy for each anatomic location. Negative results can be used to exclude PE patients with contraindications to iodine contrast or those with hemodynamic instability in the absence of other imaging modalities. However, this does not mean that non-contrast scans can replace enhanced examination. Pulmonary artery enhancement to confirm the presence of PE is indispensable. ED/OED images could identify fresh blood clots, whereas placing non acute and aged embolisms is challenging. In addition, ED and OED images do not allow for the identification of other types of thrombi, fat emboli, and tumor emboli. Any type of PE can cause symptoms, such as dyspnea, hypoxemia, and chest pain in patients [29]. Moreover, contrast-enhanced CTPAs do not just rule in/out PEs but also help us confidently convey information on the thrombo-embolic burden, aetiologies, and complications of confirmed PEs, in addition to other pulmonary artery related pathologies (sarcoma), chronic thromboembolic pulmonary hypertension [30], and mimics of PEs. Even though ED/OED predicts thrombosis, the question remains as to how to rationalise the use of ED/OED images results to help decide to perform CTPA to confirm the diagnosis of acute, sub-acute, and chronic PE, to avoid overestimation, and to maintain good clinical practice.

In this study, a quantitative assessment of non-enhanced images was carried out to account for differences in EDW values between individuals. The research identified that whether it was main pulmonary, lobar, or segmental arteries with blood clots, EDW values ranged from 108.1-108.8%, which were 3.9-4.2%EDW higher than those of the main pulmonary arteries without emboli. Meanwhile, using pulmonary arteries without emboli as a baseline (100%EDW), pulmonary arteries with emboli standardised ED values were 103.6-104.3%EDW. The findings reported contributed to our understanding of quantitative results of thrombus water content at different times, which we can use to further increase diagnostic confidence when subjective assessment is confusing. Blood clot visualization *via* ED/OED images of unenhanced chest CT is associated with the time of thrombus formation, the water content of the blood clots, or a combination of both. In clinical practice, long standing embolisms or embolisms that have been treated tend to be smaller than when first detected and will not appear hyperdense on ED images due to decreased blood clot water content, resulting in a missing diagnosis of PE. Theoretically, this may offer the possibility of distinguishing an existing *versus* a new thrombus. Furthermore, while an increase in D-dimer *via* blood analysis often indicates the non-specific possibility of thrombus formation, combining these results with a positive PE on non-contrast ED/OED images largely indicates that a thrombus has occurred.

ED/OED images usually correlate with tissue physical density. Calcium foci and calcified lymph nodes in the hilar or mediastinal region near the pulmonary arteries tend to be hyperdense, leading to hyperdense ED or OED images in or near the pulmonary arteries and affecting diagnosis. High-density pulmonary shadows on ED images often present only within the lumen of the pulmonary artery and less frequently outside the contour of the pulmonary artery, whereas high-density shadows due to calcification usually protrude outside the contour of the pulmonary artery.

## 5 LIMITATIONS

This study has several limitations. First, this was a prospective study with a relatively small study population. A larger, prospective, multicentre study is needed to confirm our findings. Second, conventional unenhanced images were not analyzed in this study; however, adjusting the narrow window setting poorly displayed central type thrombi, as mentioned in the previous literature. Other energy spectral images from unenhanced SBI, *i.e*., VMI and effective atomic number images, which may be useful in the diagnosis of PE, were not further investigated. Third, when patients were enrolled, patients with large pleural effusion or previous surgery were excluded. Those may be patients with potentially relevant pathologies that could cause PE. To some extent, there was sample selection bias. Fourth, radiological diagnoses can be affected by calcifications in the mediastinum and parapne-umonic areas. Radiologists who lack experience with exposure to ED and OED images require additional training to detect thrombi in ED and OED reconstructions to avoid incorrect diagnoses.

## CONCLUSION

In conclusion, this study determined that electron density and overlay electron density images without contrast media using SDCT could improve the visualization of PE with high sensitivity, specificity, and diagnostic accuracy. Unenhanced CT is sensitive and specific enough to accurately detect PE, whether for pulmonary artery trunks, lobar arteries, or segmental arteries. As a predictive thrombus modality, high density on ED images accurately indicates thrombus presence, which can be further confirmed using contrast. However, PE can be excluded in patients with contraindications to the contrast medium due to the high negative predictive value. ED and OED images on SDCT are valuable for PE pre evaluation in emergencies. Future studies could employ higher resolution scanning protocols to evaluate this technology better.

## Figures and Tables

**Table 1:** The precise location and total number of blood clots in 40 patients.

Location of Thrombosis	Number	Location of Thrombosis	Number
Main pulmonary artery	3	-	-
Right pulmonary artery	6	Left pulmonary artery	8
Right upper lobe artery	7	Left upper lobe artery	9
Apical segmental artery (RA1)	10	Apical segmental pulmonary artery (LA1)	10
Posterior segmental artery (RA2)	5	Posterior segmental artery (LA2)	8
Anterior segmental artery (RA3)	15	Anterior segmental artery (LA3)	14
Intermedius pulmonary artery	4	-	-
Right middle lobe artery	14	Left lingula lobe artery	9
Lateral segment artery (RA4)	7	Superior lingula artery(LA4)	7
Medial segment artery (RA5)	10	Inferior lingula artery (LA5)	11
Right lower lobe artery	15	Left lower lobe artery	11
Superior segmental artery (RA6)	9	Superior segmental artery (LA6)	10
Medial basal segmental artery (RA7)	10	Medial basal segmental artery (LA7)	15
Anterior basal segmental artery (RA8)	13	Anterior basal segmental artery (LA8)	16
Lateral basal segmental artery (RA9)	16	Lateral basal segmental artery (LA9)	12
Posterior basal segmental artery (RA10)	23	Posterior basal segmental artery (LA10)	15

**Table 2:** Summary of true positive/negative and false positive/negative results for ED and overlay ED images.

-	Individual		PAT		Lobular Artery		Pulmonary Segment Artery	
-	ED	OED	ED	OED	ED	OED	ED	OED
True positive	36	38	15	16	62	64	178	201
False positive	17	8	13	17	27	30	25	25
True negative	24	31	207	203	457	454	1327	1320
False negative	2	2	3	1	7	5	57	35

**Note: **PAT, Pulmonary Artery Trunks, including main pulmonary artery, right and left pulmonary artery. ED, Electron Density; OED, Overlay Electron Density.

**Table 3:** The value of electron density images and combined overlay electron density images for the detection of pulmonary embolisms using SDCT (95% CI).

-	Detection of PE			
	Individual	PAT	Lobular Artery	Pulmonary Segment Artery
ED	-	-	-	-
Sensitivity	94.7% (95% CI:80.9–99.1%)	83.3% (95% CI:57.7–95.6%)	89.9% (95% CI:79.6–95.5%)	75.7% (95% CI:69.7–81.0%)
Specificity	58.5% (95% CI:42.2–73.3%)	94.1% (95% CI:89.9–96.7%)	94.4% (95% CI:91.9–96.2%)	98.2% (95% CI:97.2–98.8%)
PPV	67.9% (95% CI:65.4–84.1%)	53.6% (95% CI:34.2–72.0%)	67.9% (95% CI:65.4–84.1%)	87.7% (95% CI:82.2–91.7%)
NPV	92.3% (95% CI:42.2–73.3%)	92.3% (95% CI:42.2–73.3%)	69.7% (95% CI:58.9–78.7%)	95.9% (95% CI:94.7–96.8%)
Accuracy	76.0% (95% CI:65.4–84.1%)	98.6% (95% CI:95.5–99.6%)	98.5% (95% CI:96.8–99.3%)	94.8% (95% CI:93.6–95.8%)
OED	-	-	-	-
Sensitivity	95.0% (95% CI:81.8–99.1%)	94.1% (95% CI:69.2–99.7%)	92.8% (95% CI:83.2–97.3%)	95.2% (95% CI:79.8–89.3%)
Specificity	80.0% (95% CI:63.9–90.4%)	92.2% (95% CI:87.7–95.3%)	93.8% (95% CI:91.2–95.7%)	98.1% (95% CI:97.2–98.8%)
PPV	82.6% (95% CI:68.0–91.7%)	48.5% (95% CI:31.2–66.1%)	68.1% (95% CI:57.6–77.1%)	88.9% (95% CI:83.9–92.6%)
NPV	94.1% (95% CI:78.9–99.0%)	99.5% (95% CI:96.8–100.0%)	98.9% (95% CI:97.3–99.6%)	97.4% (95% CI:96.4–98.2%)
Accuracy	87.3% (95% CI:78.1–93.2%)	92.4% (95% CI:88.3–95.2%)	93.7% (95% CI:91.3–95.9%)	96.2% (95% CI:95.1–97.1%)

**Note: **PE, Pulmonary Embolism. PAT, Pulmonary Artery Trunks, including the main pulmonary artery and right and left pulmonary arteries. PPV, Positive Predictive Value; NPV, Negative Predictive Value. ED, Electron Density; OED, Overlay Electron Density. CI Confidence Interval.

**Table 4:** Quantitative analysis of pulmonary thrombosis.

-	EDW (%)	∆ED	NED
MPA	104.1 ± 0.7	-	-
PAT	108.4 ± 3.8*	4.0 ± 3.7^#^	103.8 ± 3.5^#^
Lobular artery	108.8 ± 3.3*	4.2 ± 3.7^#^	104.3 ± 3.6^#^
Pulmonary segment artery	108.1 ± 4.0*	3.9 ± 4.1^#^	103.6 ± 3.9*

**Note: **Data are means ± standard deviations. ED, Electron Density; ∆ED, relative electron density difference; NED, Normalized Electron Density. MPA, Main Pulmonary Artery. PAT, Pulmonary Artery Trunk. *P<0.05, EDW (%) paired t tests, MPA *vs*. PAT, MPA *vs*. lobular artery, and MPA *vs*. pulmonary segment artery. #*P*>0.05, ΔED paired t tests, PAT *vs*. lobular artery, PAT *vs*. pulmonary segment artery, lobular artery *vs*. pulmonary segment artery. #*P* >0.05, NED paired t tests, PAT *vs*. lobular artery, PAT *vs*. pulmonary segment artery. *P<0.05, lobular artery *vs*. pulmonary segment artery.

## Data Availability

All data generated or analysed during this study are included in this published article.

## LIST OF ABBREVIATIONS

ED: Electron Density
OED: Overlay Electron Density
PE: Pulmonary Embolism
APE: Acute Pulmonary Embolism
SDCT: Spectral Detector CT
CTPA: Computed Tomography Pulmonary Angiography
PPV: Positive Predictive Value
NPV: Negative Predictive Value

## STANDARDS OF REPORTING

STROBE guidelines were followed.

## References

[1] Wendelboe A.M., Raskob G.E. (2016). Global burden of thrombosis: Epidemiologic aspects.. Circ. Res..

[2] Raskob G.E., Angchaisuksiri P., Blanco A.N., Buller H., Gallus A., Hunt B.J., Hylek E.M., Kakkar A., Konstantinides S.V., McCumber M., Ozaki Y., Wendelboe A., Weitz J.I. (2014). Thrombosis: A major contributor to global disease burden.. Arterioscler. Thromb. Vasc. Biol..

[3] Harvey J.J., Huang S., Uberoi R. (2022). Catheter-directed therapies for the treatment of high risk (massive) and intermediate risk (submassive) acute pulmonary embolism.. Cochrane Database Syst. Rev..

[4] Barco S., Valerio L., Gallo A., Turatti G., Mahmoudpour S.H., Ageno W., Castellucci L.A., Cesarman-Maus G., Ddungu H., De Paula E.V., Dumantepe M., Goldhaber S.Z., Guillermo Esposito M.C., Klok F.A., Kucher N., McLintock C., Ní Áinle F., Simioni P., Spirk D., Spyropoulos A.C., Urano T., Zhai Z., Hunt B.J., Konstantinides S.V. (2021). Global reporting of pulmonary embolism–related deaths in the World Health Organization mortality database: Vital registration data from 123 countries.. Res. Pract. Thromb. Haemost..

[5] Zantonelli G., Cozzi D., Bindi A., Cavigli E., Moroni C., Luvarà S., Grazzini G., Danti G., Granata V., Miele V. (2022). Acute pulmonary embolism: Prognostic role of Computed Tomography Pulmonary Angiography (CTPA).. Tomography.

[6] Kline J.A., Garrett J.S., Sarmiento E.J., Strachan C.C., Courtney D.M. (2020). Over-testing for suspected pulmonary embolism in american emergency departments.. Circ. Cardiovasc. Qual. Outcomes.

[7] Raji H., Javad Moosavi S.A., Dastoorpoor M., Mohamadipour Z., Mousavi Ghanavati P. (2018). Overuse and underuse of pulmonary CT angiography in patients with suspected pulmonary embolism.. Med. J. Islam. Repub. Iran.

[8] Mirabile A., Lucarelli N.M., Sollazzo E.P., Stabile Ianora A.A., Sardaro A., Mirabile G., Lorusso F., Racanelli V., Maggialetti N., Scardapane A. (2021). CT pulmonary angiography appropriateness in a single emergency department: Does the use of revised Geneva score matter?. Radiol. Med. (Torino).

[9] Ehsanbakhsh A., Hatami F., Valizadeh N., Khorashadizadeh N., Norouzirad F. (2021). Evaluating the performance of unenhanced computed tomography in the diagnosis of pulmonary embolism.. J Tehran Heart Cent.

[10] Franco P.N., Spasiano C.M., Maino C., De Ponti E., Ragusi M., Giandola T., Terrani S., Peroni M., Corso R., Ippolito D. (2023). Principles and applications of dual-layer spectral CT in gastrointestinal imaging.. Diagnostics (Basel).

[11] Rassouli N., Etesami M., Dhanantwari A., Rajiah P. (2017). Detector-based spectral CT with a novel dual-layer technology: Principles and applications.. Insights Imaging.

[12] Nagayama Y., Inoue T., Oda S., Tanoue S., Nakaura T., Morinaga J., Ikeda O., Hirai T. (2021). Unenhanced dual-layer spectral-detector CT for characterizing indeterminate adrenal lesions.. Radiology.

[13] Yu Y., Fu Y., Chen X., Zhang Y., Zhang F., Li X., Zhao X., Cheng J., Wu H. (2022). Dual-layer spectral detector CT: Predicting the invasiveness of pure ground-glass adenocarcinoma.. Clin. Radiol..

[14] Lai L.Y., Jiang Y., Shu J. (2023). The application of dual-layer spectral detector CT in abdominal vascular imaging.. Curr. Med. Imaging.

[15] Daoud B., Cazejust J., Tavolaro S., Durand S., Pommier R., Hamrouni A., Bornet G. (2021). Could spectral CT have a potential benefit in coronavirus disease (COVID-19)?. AJR Am. J. Roentgenol..

[16] Sedaghat S., Langguth P., Larsen N., Campbell G., Both M., Jansen O. (2021). Diagnostic accuracy of dual-layer spectral CT using electron density images to detect post-traumatic prevertebral hematoma of the cervical spine.. Röfo Fortschr. Geb. Röntgenstr. Neuen Bildgeb. Verfahr..

[17] Bae K., Jeon K.N. (2021). Diagnosis of pulmonary embolism in unenhanced dual energy CT using an electron density image.. Diagnostics (Basel).

[18] Cham M.D., Yankelevitz D.F., Shaham D., Shah A.A., Sherman L., Lewis A., Rademaker J., Pearson G., Choi J., Wolff W., Prabhu P.M., Galanski M., Clark R.A., Sostman H.D., Henschke C.I. (2000). Deep venous thrombosis: Detection by using indirect CT venography.. Radiology.

[19] Fehrenbach U., Feldhaus F., Kahn J., Böning G., Maurer M.H., Renz D., Frost N., Streitparth F. (2019). Tumour response in non‐small‐cell lung cancer patients treated with chemoradiotherapy – Can spectral CT predict recurrence?. J. Med. Imaging Radiat. Oncol..

[20] Ghasemi Shayan R., Oladghaffari M., Sajjadian F., Fazel Ghaziyani M. (2020). Image quality and dose comparison of single-energy CT (SECT) and dual-energy CT (DECT).. Radiol. Res. Pract..

[21] Bogot N.R., Fingerle A., Shaham D., Nissenbaum I., Sosna J. (2011). Image quality of low-energy pulmonary CT angiography: Comparison with standard CT.. AJR Am. J. Roentgenol..

[22] Sun S., Semionov A., Xie X., Kosiuk J., Mesurolle B. (2014). Detection of central pulmonary embolism on non-contrast computed tomography: A case control study.. Int. J. Cardiovasc. Imaging.

[23] Tatco V.R., Piedad H.H. (2011). The validity of hyperdense lumen sign in non-contrast chest CT scans in the detection of pulmonary thromboembolism.. Int. J. Cardiovasc. Imaging.

[24] Ehn S., Sellerer T., Muenzel D., Fingerle A.A., Kopp F., Duda M., Mei K., Renger B., Herzen J., Dangelmaier J., Schwaiger B.J., Sauter A., Riederer I., Renz M., Braren R., Rummeny E.J., Pfeiffer F., Noël P.B. (2018). Assessment of quantification accuracy and image quality of a full‐body dual‐layer spectral CT system.. J. Appl. Clin. Med. Phys..

[25] Neuhaus V., Lennartz S., Abdullayev N., Große Hokamp N., Shapira N., Kafri G., Holz J.A., Krug B., Hellmich M., Maintz D., Borggrefe J. (2018). Bone marrow edema in traumatic vertebral compression fractures: Diagnostic accuracy of dual-layer detector CT using calcium suppressed images.. Eur. J. Radiol..

[26] So A., Nicolaou S. (2021). Spectral computed tomography: Fundamental principles and recent developments.. Korean J. Radiol..

[27] Mei K., Ehn S., Oechsner M., Kopp F.K., Pfeiffer D., Fingerle A.A., Pfeiffer F., Combs S.E., Wilkens J.J., Rummeny E.J., Noël P.B. (2018). Dual-layer spectral computed tomography: Measuring relative electron density.. Eur. Radiol. Exp..

[28] Nelles C., Lennartz S. (2023). Spinal hematoma visualized with dual-energy CT-derived electron density overlay maps.. Radiology.

[29] Konstantinides S.V., Meyer G., Becattini C., Bueno H., Geersing G.J., Harjola V.P., Huisman M.V., Humbert M., Jennings C.S., Jiménez D., Kucher N., Lang I.M., Lankeit M., Lorusso R., Mazzolai L., Meneveau N., Áinle F.N., Prandoni P., Pruszczyk P., Righini M., Torbicki A., Van Belle E., Zamorano J.L. (2019). 2019 ESC Guidelines for the diagnosis and management of acute pulmonary embolism developed in collaboration with the European Respiratory Society (ERS).. Eur. Respir. J..

[30] Yan L., Li X., Liu Z., Zhao Z., Luo Q., Zhao Q., Jin Q., Yu X., Zhang Y. (2019). Research progress on the pathogenesis of CTEPH.. Heart Fail. Rev..

